# Preparation and Characterization of Functionalized Graphene Oxide Carrier for siRNA Delivery

**DOI:** 10.3390/ijms19103202

**Published:** 2018-10-17

**Authors:** Jing Li, Xu Ge, Chunying Cui, Yifan Zhang, Yifan Wang, Xiaoli Wang, Qi Sun

**Affiliations:** 1 Department of Pharmaceutics, School of Pharmaceutical Sciences, Capital Medical University, Beijing 100069, China; lijingcr7@foxmail.com (J.L.); gexu1991@foxmail.com (X.G.); zyfemily@163.com (Y.Z.); wyf19950527@126.com (Y.W.); 15810587759@163.com (X.W.); 15801371767@163.com (Q.S.); 2 Engineering Research Center of Endogenous Prophylactic of Ministry of Education of China, Beijing 100069, China; 3 Beijing Area Major Laboratory of Peptide and Small Molecular Drugs, Beijing 100069, China

**Keywords:** graphene oxide (GO), *VEGF-siRNA*, small interfering RNA, gene delivery, nanocarrier

## Abstract

A successful siRNA delivery system is dependent on the development of a good siRNA carrier. Graphene oxide (GO) has gained great attention as a promising nanocarrier in recent years. It has been reported that GO could be used to deliver a series of drugs including synthetic compounds, proteins, antibodies, and genes. Our previous research indicated that functionalized GO could deliver siRNA into tumor cells and induce a gene silencing effect, to follow up the research, in this research, GO-R8/cRGDfV(GRcR) was designed and prepared for *VEGF-siRNA* delivery as a novel carrier. The Zeta potential and particle size of the new designed GRcR carrier was measured at (29.46 ± 5.32) mV and (135.7 ± 3.3) nm respectively, and after transfection, the VEGF mRNA level and protein expression level were down-regulated by 48.22% (*p* < 0.01) and 38.3% (*p* < 0.01) in HeLa cells, respectively. The fluorescent images of the treated BALB/c nude mice revealed that GRcR/*VEGF-siRNA* could conduct targeted delivery of *VEGF-siRNA* into tumor tissues and showed a gene silencing effect as well as a tumor growth inhibitory effect (*p* < 0.01) in vivo. Further studies showed that GRcR/*VEGF-siRNA* could effectively inhibit angiogenesis by suppressing VEGF expression. Histology and immunohistochemistry studies demonstrated that GRcR/*VEGF-siRNA* could inhibit tumor tissue growth effectively and have anti-angiogenesis activity, which was the result of VEGF protein downregulation. Both in vitro and in vivo results demonstrated that GRcR/*VEGF-siRNA* could be used as an ideal nonviral tumor-targeting vector for *VEGF-siRNA* delivery in gene therapy.

## 1. Introduction

RNA interference (RNAi) is a powerful approach and a promising technology used in gene therapy. It has been used to treat a variety of genetic diseases through inhibiting specific disease-causing gene function [1,2,3,4]. Nowadays, RNAi has been used in many therapeutic treatments, including clinical trials such as the treatment of age-related macular degeneration and respiratory syncytial virus [5]. However, naked siRNA delivery is still limited due to these characters: poor water-solubility and high molecular weight [6,7,8].

Lately, graphene oxide (GO) has gained attention due to its unique characteristics including good water dispensability, large surface area, and good biocompatibility. It has been used to design carriers to load therapeutic drugs, proteins, antibodies, and DNA and RNA segments by π–π* stacking, electrostatic attraction, and other molecular interactions [9,10,11,12,13]. Some studies showed that GO could be used to deliver nucleic acids in gene silencing treatments. For instance, Tripathi et al. [14] developed a functionalized GO of linear polyethylenimine-grafted graphene oxide (LP-GO) which could be used as an effective carrier in cell transfection. Feng et al. [15] prepared an ultrasmall dual-polymer-functionalized GO (GO-PEG-PEI) which could be transfected siRNA into cells and downregulate the expression of target genes like Polo-like kinase 1 (Plk 1). Yang et al. [16] modified GO with polyethylene glycol (PEG) and folic acid to deliver *hTERT-siRNA* into cancer cells. It could target HeLa cells and silence the relative mRNA expression.

Cell penetrating peptides (CPPS) are a family of functional peptides that mediate the entry of bioactive substances into cells. CPPS can transfer drugs, genes, and large molecules intracellularly. Octaarginine (R8) belongs to the CPPS family, and has been widely reported and used as a nanogene carrier. It is positively charged and can enhance cellular uptake because of its strong cell adhesive mechanism. Yukawa et al. [17] used R8 to label quantum dots (QD) which could be successfully used in adipose tissue-derived stem cell (ASC) imaging. Kamei et al. [18] reported that the safety and effectiveness of the intestinal absorption of protein drugs could be enhanced after the drug surface is modified with R8. While Golan et al. [19] synthesized FITC-labeled HPMA (hydrophilic *N*-(2-hydroxypropyl) methacrylamide) copolymer-bound R8 (P-R8-FITC), and after conjugation with the HA2 fusogenic peptide, P-(R8)-(HA2)-FITC was delivered and could decreased the target mRNA level significantly. An intergrin-mediated polypeptide, cyclic RGD peptides (cRGD), is frequently used in tumor-targeted therapies [20,21]. Wang et al. [22,23] modified nanocarriers with cRGD and successfully improved the targeting and transfection efficiency. Allman et al. [24] reported that cRGDfV has angiogenesis inhibitory activity and has a synergistic function with *VEGF-siRNA* to inhibit angiogenesis.

**Scheme 1:**
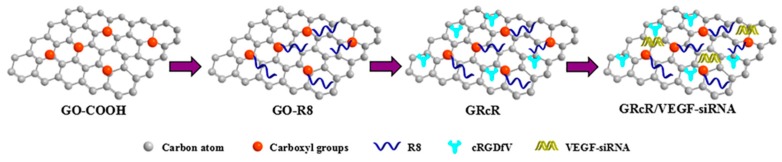
Schematic diagram of the preparation of the GRcR/VEGF-small interfering RNA (siRNA).

Our previous research indicated that functionalized GO can deliver siRNA into HeLa cells and induce gene silencing effect, and in this study, a nanogene delivery system was designed and prepared using R8 and cRGDfV-functionalized GO to increase the electropositivity and transfection efficiency [25,26,27] (Scheme 1). The results demonstrated that *VEGF-siRNA* was delivered into HeLa cells in the form of GRcR/*VEGF-siRNA* and could downregulate the expression of the *VEGF* gene. The tumor growth investigation was carried out both in vitro and in vivo.

## 2. Results

### 2.1. Characterization (Fourier Transform Infrared Spectroscopy (FTIR), Ultraviolet Visible Spectrophotometer (UV), and Transmission Electron Microscope (TEM))

**Figure 1:**
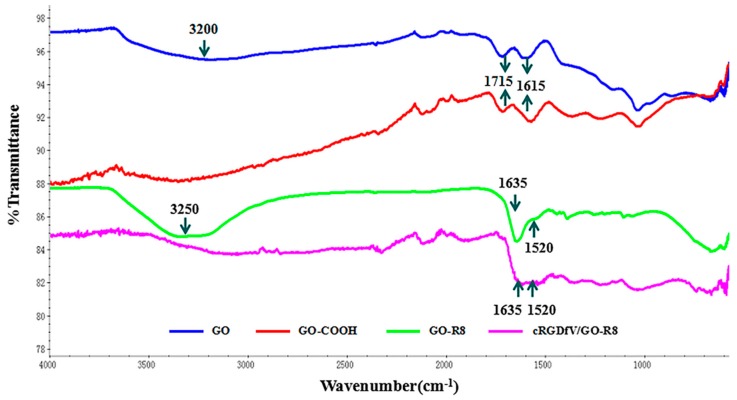
Fourier transform infrared spectroscopy (FTIR) spectra of graphene oxide (GO) (blue), GO-COOH (red), GR (green), and GRcR (purple).

The structural elucidation of GO, GO-COOH, GR, and GRcR was carried out using FTIR and UV spectroscopy. In FTIR spectrum, blue curve of GO showed multiple stretching vibration peaks around ~3200, ~1715 and ~1615 cm^−1^, which are assigned to O-H, C=O and C=C respectively. After modification, amide I and amide II peak at ~1635 and ~1520 cm^−1^ appeared in the GR curve (green) were indicated the successful linking of R8. After the modification of cRGDfV, both the above peaks linked together to become a plump peak (purple), which indicated the successful modification of GR (Figure 1).

**Figure 2:**
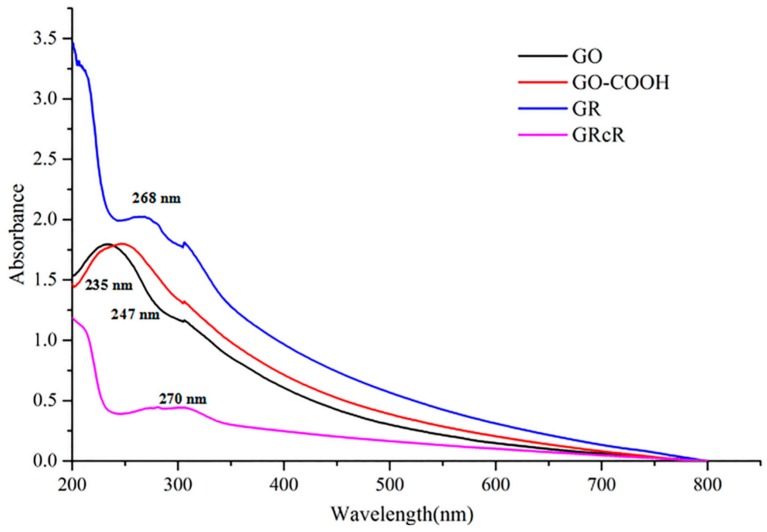
The UV–Vis spectra of GO (black), GO-COOH (red), GR (blue), and GRcR (purple).

The functionalized GO was further characterized by UV–Vis spectroscopy. As shown in Figure 2, the absorption peak of GO at 235 nm indicates the π–π* transition of the C=C bond. Compared to GO, the GO-COOH peak had a red-shift to 247 nm which was caused by inducing the carboxyl group. The absorption peak for GR was observed at 268 nm which showed a red-shift compared with GO-COOH. In the UV–Vis spectrum of GRcR, the absorption peak was observed at 270 nm, due to the decreased π–π* conjugation by cRGDfV modification.

**Figure 3:**
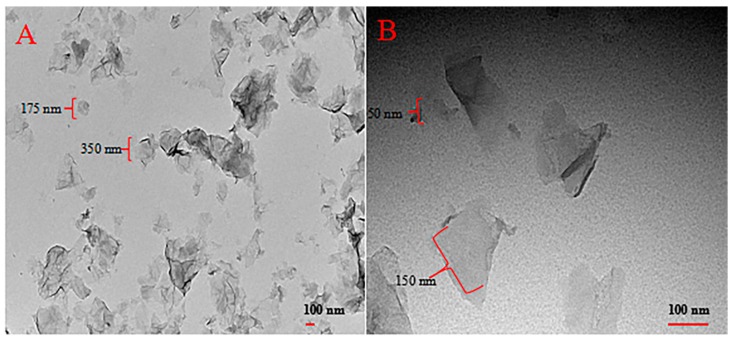
Transmission electron microscopy (TEM) images of the GO (**A**) and GRcR (**B**). (Scale bar: 100 nm).

Morphology studies of GRcR were carried out using transmission electron microscopy (TEM). In TEM images, as shown in Figure 3A, GO was a wrinkle paper-like sheet with diameter 100 to 350 nm. After being modified with R8 and cRGDfV, the diameter of the complex was reduced to 50 to 150 nm; the reduction was possibly caused by the improvement of dispensability and hydrophilicity.

### 2.2. Tyndall Effect, Zeta Potential, Stability, and Particle Size Studies

**Figure 4:**
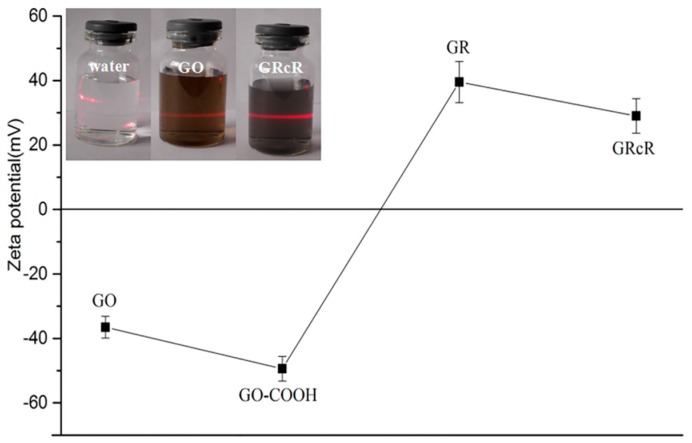
Tyndall effect and Zeta potential of GO, GO-COOH, GR, and GRcR. The data is presented as the mean ± SD, *n* = 3.

As shown in Figure 4, the Tyndall effect was observed, indicating that the GRcR solution was an anano-colloidal disperse system. Water and GO suspensions were used as controls.

Zeta potentials of GO, GO-COOH, GR, and GRcR were obtained; the results are shown in Figure 4. GO was negatively charged at (−36.51 ± 3.38) mV and after carboxylation, the zeta potential of GO-COOH was changed to (−49.41 ± 3.84) mV. However, the electrical properties of the particle dramatically changed after the particle was R8-modified, the zeta potential was measured at (39.54 ± 6.43) mV, which illustrated that the R8 was successfully connected to GO. After the structure with was cRGDfV-modified, the zeta potential of GRcR had a mild decrease to 29 ± 5.32 mV, but the complex was still positively charged.

**Figure 5:**
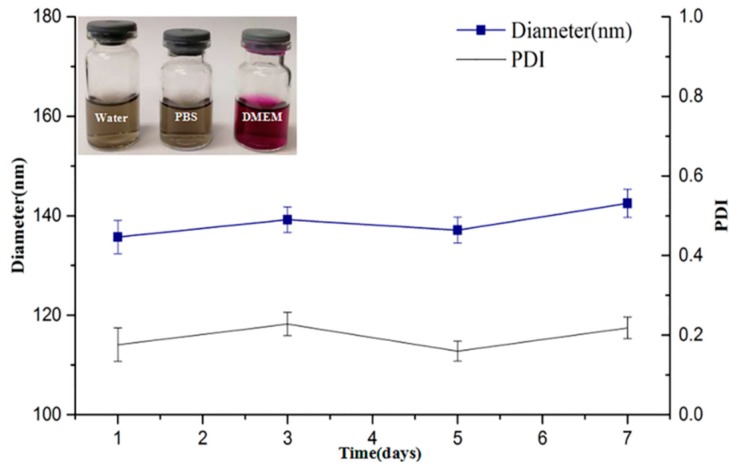
Dispensability, stability, particle size, and PDI (polydispersity index) diagram of GRcR. Solvent: water. Duration: seven days. The data is presented as the mean ± SD, *n* = 3.

The stability of the GRcR solution was studied. Freeze-dried GRcR was suspended into water, PBS (phosphate buffered saline), and cell culture medium DMEM (Dulbecco’s modified Eagle’s medium), respectively, and left at room temperature. After seven days of observation, no flocculation was found and, in order to investigate the stability of GRcR solution, particle size and PDI (polydispersity index) were measured in water. As shown in Figure 5, the particle size and PDI of GRcR were approximately 140 and 0.2 nm, respectively. The stability results showed that GRcR has good stability in water after seven days at room temperature.

### 2.3. Differential Scanning Calorimeter (DSC) and Thermal Gravimetric Analyzer (TGA) Spectra

**Figure 6:**
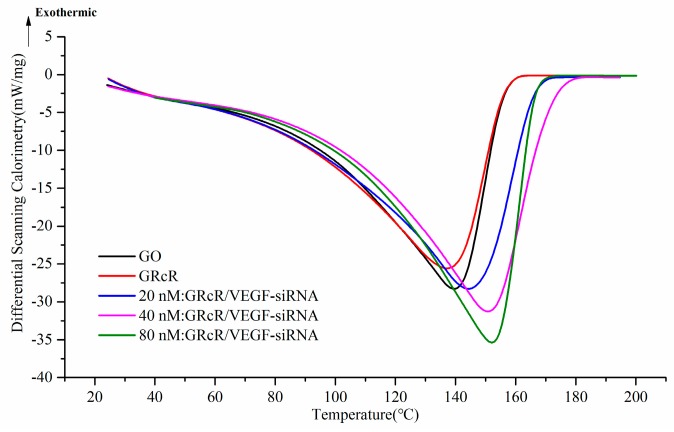
Differential scanning calorimeter (DSC) spectra of GO (black), GRcR (red), and GRcR loaded with different concentrations of *VEGF-siRNA* (20, 40 and 80 nM).

Thermal kinetics parameters of GO and GRcR were characterized by DSC. GRcR was loaded with different concentrations of *VEGF-siRNA* from 20 to 80 nM. The endothermic peaks changed and the *VEGF-siRNA* concentration increased (Figure 6, blue, purple, and green curve), which suggested that *VEGF-siRNA* was successfully loaded onto GRcR.

**Figure 7:**
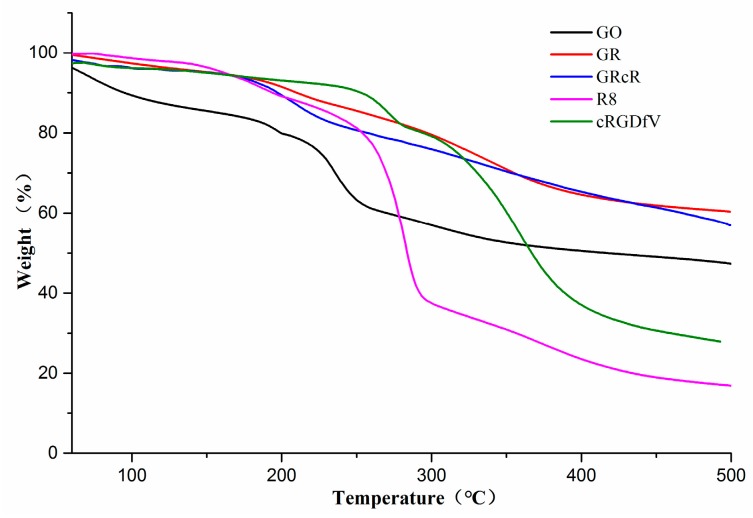
Thermal gravimetric analyzer (TGA) spectra of GO (black), GR (red), GRcR (blue), R8 (purple), and cRGDfV (green).

The thermal gravimetric parameters of GO, GR, and GRcR were analyzed using TGA. Results are shown in Figure 7, the weight of GO (black curve) started to decrease with increasing temperature, which was attributed to solvent evaporation; a major mass loss occurred at 200 °C, and was caused by the pyrolysis of the labile oxygen-containing group. For GR and GRcR, no major mass loss was observed and, during the test period, the weight of these complexes was stable and continuous. The percentage weight loss (Delta Y) of GO, R8, and cRGDfV was 33.55%, 60.70%, and 65.23%, respectively, while, the weight loss of GO-R8 and GRcR was 37.16% and 37.67%, respectively. The composition of the complex could be calculated using the weight loss data above; it contains approximately 85.04 wt % GO, 13.05 wt % R8, and 1.91 wt % cRGDfV.

### 2.4. Gel Retardation Assay

**Figure 8:**
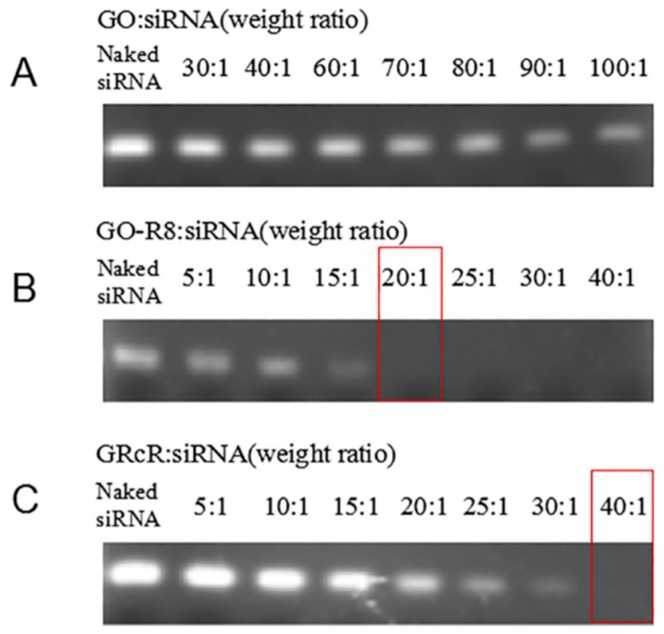
Agarose gel retardation assay of *VEGF-siRNA* loaded complexed. (**A**) GO/*VEGF-siRNA*. (**B**) GO-R8/*VEGF-siRNA*. (**C**) GRCR/*VEGF-siRNA*. The red frame represents the best proportion.

The loading capability of GRcR was evaluated by gel retardation assay. Naked *VEGF-siRNA* served as negative control, and a series of GRcR loaded with *VEGF-siRNA* at different ratios (*w/w*, 5:1, 10:1, 15:1, 20:1, 25:1, 30:1, 40:1) were prepared as test candidates, the results are shown in Figure 8. RNA bands in the GO/*VEGF-siRNA* group could all be detected at all test ratios, which illustrated that the GO could not be used as a gene carrier at the selected concentration. In Figure 8B, bands disappeared at the ratio of 20:1 in the GO-R8/*VEGF-siRNA* group. After being modified with the cRGD group (Figure 8C), the bright RNA band disappeared at the ratio of 40:1, which indicated that 1.0 mg of *VEGF-siRNA* could be effectively absorbed by 40 mg GRcR. According to the gel retardation results, the ratio of 40:1 was used in the following experiments.

### 2.5. Release Profile of VEGF-siRNA

**Figure 9:**
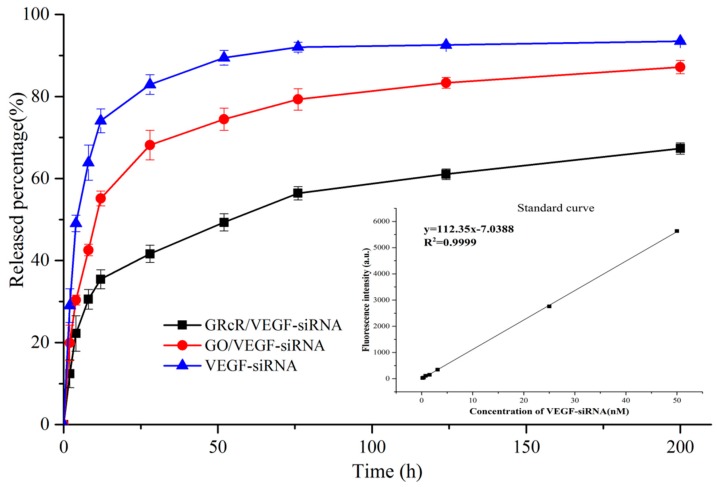
Release profile of *VEGF-siRNA* from GO (red) and GRcR (black). The data was presented as the mean ± SD, *n* = 3.

Release profile of *VEGF-siRNA* from GO and GRcR were studied using dialysis. The cumulative release curves are shown in Figure 9. In the results, only approximately 35% of *VEGF-siRNA* was released from GRcR during the first 12 h (black); while 55% of *VEGF-siRNA* was released from GO (red). After 200 h, the amounts of siRNA released from GO reached 87%, while only approximately 67% of *VEGF-siRNA* was released from GRcR. The results illustrated that the GRcR could be used as a late release carrier in a gene delivery system in vitro and, in the case of internal circulation, the nanocarrier might prolong *VEGF-siRNA* release into tumor tissues.

### 2.6. Cytotoxicity Assay

**Figure 10:**
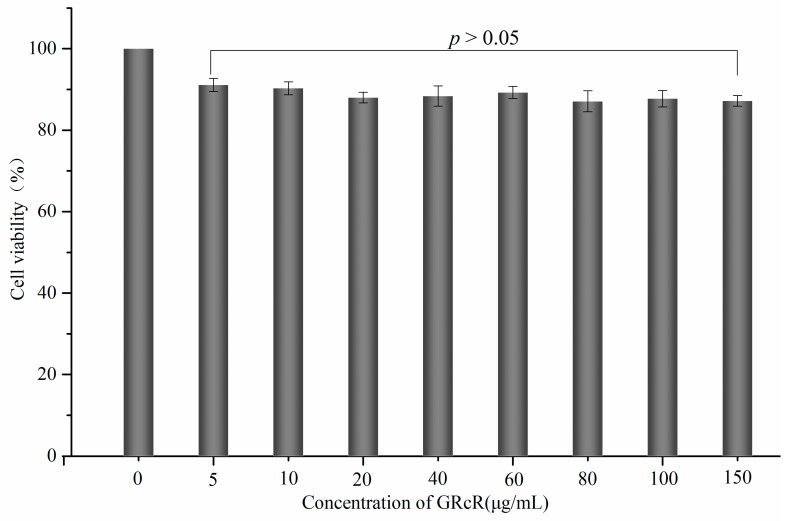
MTT assay for GRcR at different concentrations of HeLa cells, the data was presented as the mean ± SD, *n* = 3.

HeLa cells were incubated with different concentrations (5–150 µg/mL) of GRcR for 48 h. The results showed that the cell viability of the tested groups remained above 85%, which indicated that no significant difference was found between each group (Figure 10). Therefore, the results illustrated that, as a carrier, GRcR has no significant cytotoxicity and no antiproliferation inhibition at selected concentrations against HeLa cells.

### 2.7. Cellular Uptake of GRcR-VEGF-siRNA

**Figure 11:**
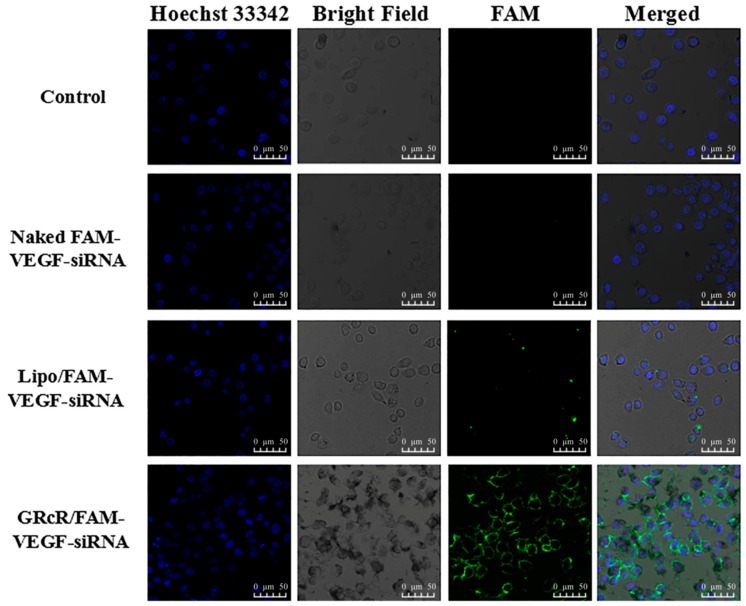
Cellular uptake of the FAM-labeled *VEGF-siRNA* in HeLa cells.

HeLa cells were transfected with naked FAM-labeled *VEGF-siRNA*, Lipo^TM^2000/FAM-labeled *VEGF-siRNA*, and GRcR/FAM-labeled *VEGF-siRNA*. Untreated HeLa cells were used as blank control. After incubating for 12 h, and transfecting for 4 h, the results were observed using a laser scanning confocal microscope (Figure 11). In the naked FAM-labeled *VEGF-siRNA* group, no green fluorescence was observed, which indicated that, without a carrier, naked siRNA had difficulty crossing the cell membrane. In the Lipo™2000/FAM-labeled *VEGF-siRNA* group (positive control), a small amount of green fluorescence was observed, indicating that the liposome successfully transfected siRNA into the cells. In the GRcR/FAM-labeled *VEGF-siRNA* group, a large amount of green fluorescence was observed, which indicated even more siRNA had been delivered. This result proved that GRcR is a better delivery agent and could effectively deliver the *VEGF-siRNA* into cells.

### 2.8. Tumor Growth Inhibition In Vitro

**Figure 12:**
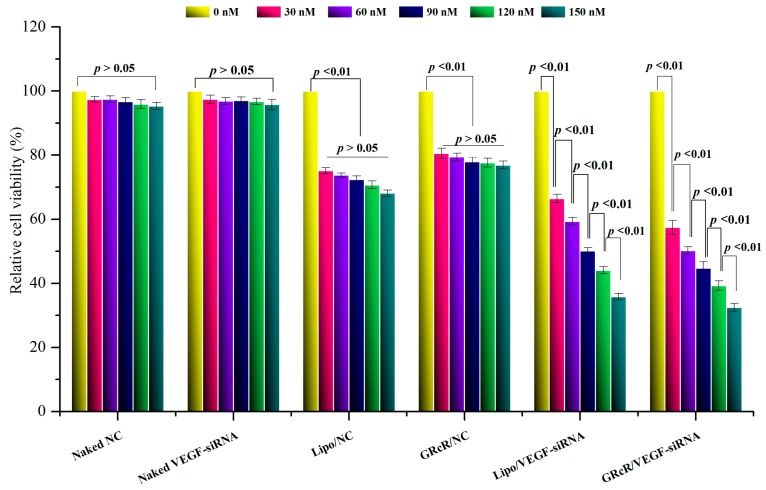
HeLa cell viability treated with NC, *VEGF-siRNA*, Lipo™2000/NC, GRcR/NC, Lipo™2000/*VEGF-siRNA*, and GRcR/*VEGF-siRNA*. The data is presented as the mean ± SD, *n* = 3.

HeLa cell viability was studied after treating with GRcR/*VEGF-siRNA*. Naked NC and naked *VEGF-siRNA* were used as blank controls, Lipo™2000/NC and GRcR/NC groups were used as negative controls, and Lipo™2000/*VEGF-siRNA* was used as a positive control (Figure 12). In the blank control groups, no significant cell viability inhibition was observed, while in the Lipo™2000/NC and GRcR/NC groups, the cell inhibition rate had no concentration-dependent effect. The inhibition rates in the Lipo™2000/*VEGF-siRNA* and GRcR/*VEGF-siRNA* groups were significantly higher than the blank and negative controls (*p* < 0.01) and were similar. With increasing concentration of siRNA, both showed concentration-dependent effects against the HeLa cells (*p* < 0.01). After considering the confocal microscope results and the tumor cell inhibitory results, GRcR/*VEGF-siRNA* was able to transfect the selected siRNA into HeLa cells and inhibit cell viability in vitro.

### 2.9. Gene Silencing Effect Evaluation on mRNA Level

**Figure 13:**
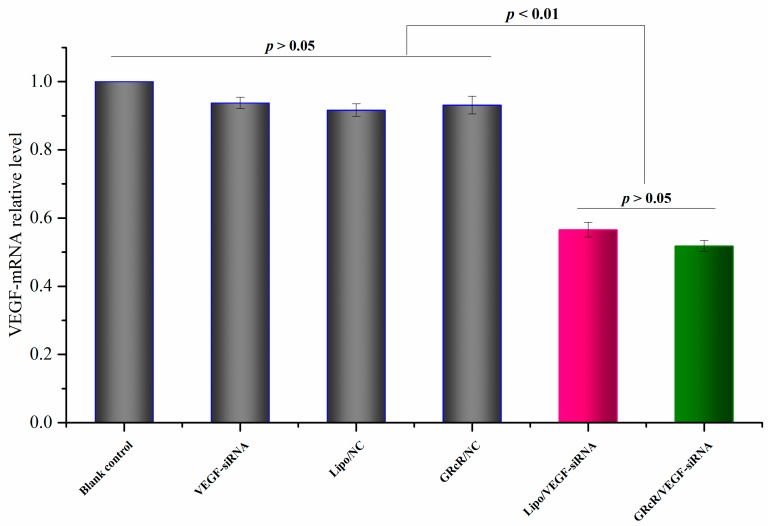
VEGF mRNA levels of the HeLa cells treated with naked *VEGF-siRNA*, Lipo™2000/NC, GRcR/NC, and *VEGF-siRNA*. The data is presented as the mean ± SD, *n* = 3.

The downregulation of mRNA was investigated by RT-PCR assay; results are shown in Figure 13. Naked *VEGF-siRNA*, Lipo™2000/NC, and GRcR/NC were used as negative controls, while Lipo™2000/*VEGF-siRNA* was used as a positive control. The result of the RT-PCR assay indicated that GRcR/*VEGF-siRNA* could significantly deliver the siRNA into cells, and downregulate the expression of VEGF mRNA by 48.22%. In the meantime, the mRNA levels in naked *VEGF-siRNA*, Lipo™2000/NC, and the GRcR/NC groups showed no significant reduction. In the Lipo™2000/*VEGF-siRNA* group, mRNA level was downregulated by 43% which was even lower than the GRcR/*VEGF-siRNA* group. The results illustrated that *VEGF-siRNA* could be delivered into cells by GRcR interfering with host mRNA causing a gene silencing effect.

### 2.10. Gene Silencing Effect Evaluation on Protein Level

**Figure 14:**
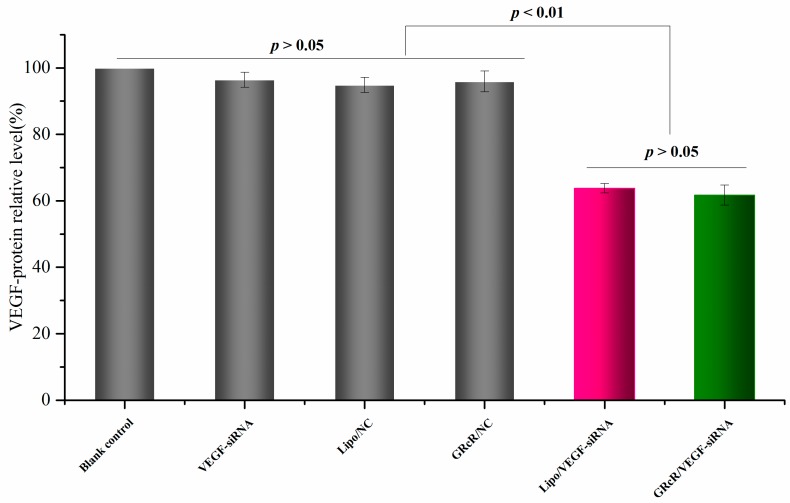
VEGF protein level of HeLa cells, treated with *VEGF-siRNA*, Lipo™2000/NC, GRcR/NC, and GRcR/*VEGF-siRNA*. The data was presented as the mean ± SD, *n* = 3.

The gene silencing efficacy of GRcR/*VEGF-siRNA* was also evaluated at the protein level, using ELISA. The VEGF level of treatment groups are shown in Figure 14, naked *VEGF-siRNA*, Lipo™2000/NC, and GRcR/NC were used as negative controls, while Lipo™2000/*VEGF-siRNA* was used as the positive control. VEGF expression in the Lipo™2000/*VEGF-siRNA* group was inhibited by 35% while, in the GRcR/*VEGF-siRNA* group, the inhibition of VEGF expression was at 38%. Compared to the negative controls, no significant inhibitory activities were observed. These results reconfirmed that GRcR/*VEGF-siRNA* could induce a gene silencing effect that could be detected at protein level.

### 2.11. Tumors Inhibition Assay In Vivo

**Figure 15:**
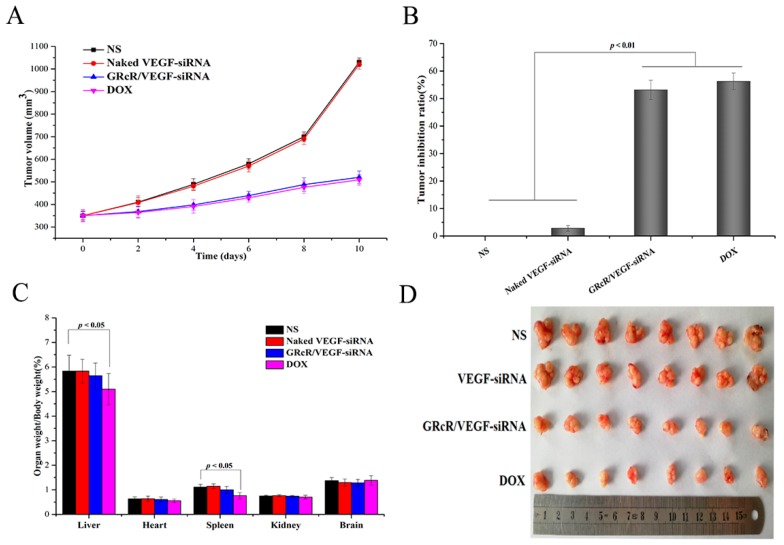
In vivo tumor growth assay. (**A**) Tumor volume. (**B**) Tumor inhibition rate of test groups. (**C**) The ratios of organ/bodyweight. (**D**) Image of tumors. The data is presented as the mean ± SD, *n* = 3.

The GRcR/*VEGF-siRNA* delivery system was studied in vivo using BALB/c nude mice. Tumor volumes of the NS and naked *VEGF-siRNA* groups were shown to have no significant difference (*p* > 0.05, Figure 15A); however, they were significantly higher than the GRcR/*VEGF-siRNA* and DOX groups (*p* < 0.01). The tumor inhibition rate was then calculated and the results showed that the rate in the GRcR/*VEGF-siRNA* and DOX groups was 53.15% and 56.28%, respectively, compared to the *VEGF-siRNA* group (2.8%) (Figure 15B). The results showed that tumor growth could be inhibited by administrating GRcR/*VEGF-siRNA* intravenously, and that the inhibitory effect was possibly caused by the gene silencing effect of the tumor cells. The ratios of organ to body weight indicated that NS, naked *VEGF-siRNA*, and GRcR/*VEGF-siRNA* groups had no tumor inhibitory activities (Figure 15C), and that the ratios were not significant different from each other. In the DOX group, the ratios of liver/body and spleen/body were reduced, and these could be caused by tissue damage impairing by doxorubicin.

### 2.12. Distribution of GRcR/VEGF-siRNA In Vivo

**Figure 16:**
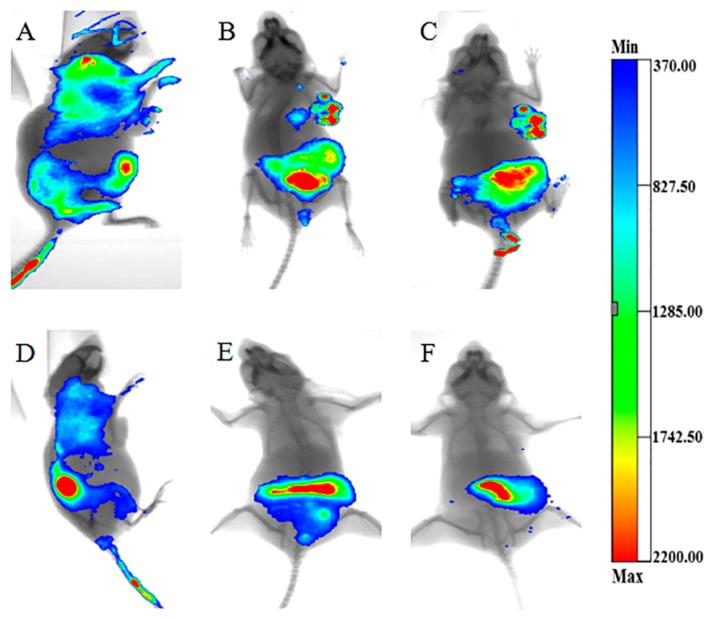
Fluorescent images of nude mice treated with GRcR/Cy5-*VEGF-siRNA*. (**A**) GRcR/Cy5-*VEGF-siRNA* at 30 min. (**B**) GRcR/Cy5-*VEGF-siRNA* at 5 h. (**C**) GRcR/Cy5-*VEGF-siRNA* at 8 h. (**D**) Cy5-*VEGF-siRNA* at 30 min. (**E**) Cy5-*VEGF-siRNA* at 5 h. (**F**) Cy5-*VEGF-siRNA* at 8 h.

The in vivo distribution profile of GRcR/*VEGF-siRNA* was studied by fluorescent imaging assay. Test results were recorded from 30 min after treatment with GRcR/Cy5-*VEGF-siRNA*. In Figure 16A–C, at 30 min, slight fluorescence could be observed in the tumor area, and after 5 h, the fluorescence in tumor area was highlighted. After 8 h, the fluorescence in the tumor area grew stronger and it could also be observed in liver, intestinal tract area, and feces. The results indicated that the drug was being metabolized and the possible excretion pathway was through the feces. In Figure 16D–F, naked Cy5-*VEGF-siRNA* was administrated, no fluorescence was observed in the tumor area, and the drug was excreted directly within 5 h. This result showed that GRcR/*VEGF-siRNA* could successfully deliver the *VEGF-siRNA* into tumor tissues in vivo, and could be used as a tumor targeting drug delivery system.

### 2.13. Inhibition of VEGF-Induced Angiogenesis

**Figure 17:**
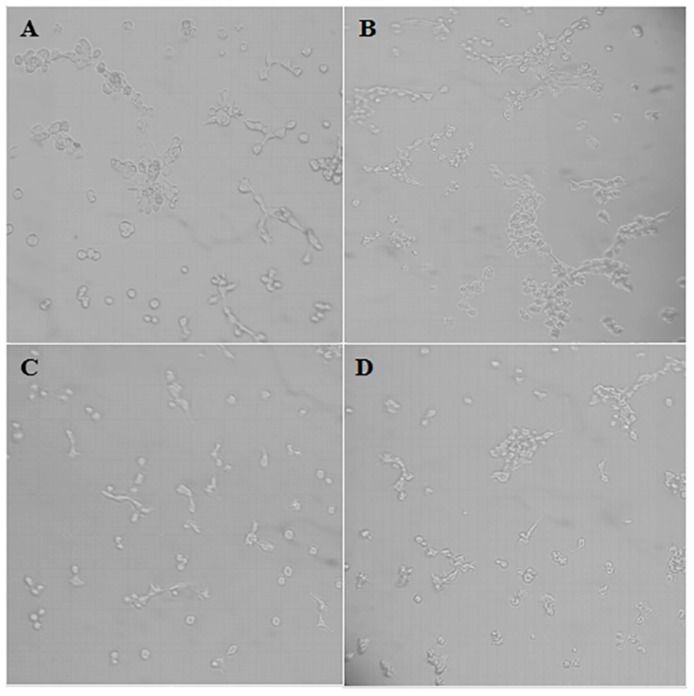
The migratory capacity of human umbilical vein endothelial cells (HUVECs). (**A**) Nontreated HUVECs, (**B**) VEGF-rich medium treated HUVECs, (**C**) GRcR/*VEGF-siRNA* treated HUVECs, and (**D**) Lipo™2000/*VEGF-siRNA* treated HUVECs. (Original magnification: 5*×*).

VEGF-induced anti-angiogenesis was studied against human umbilical vein endothelial cells (HUVECs); the result is shown in Figure 17A. HUVECs incubated with normal fresh medium were used as blank control and, after 4 h incubation, robust tubes were observed. In Figure 17B, HUVECs were cultured with VEGF-rich medium (prepared from a HeLa cell medium) and served as negative control. After 4 h incubation, an abundance of tubes could be observed clearly. In Figure 17C, few tubes formed when HUVECs were cultured with HeLa cell medium pretreated with GRcR/*VEGF-siRNA*, and the same result was also observed for the Lipo™2000/*VEGF-siRNA* test group (Figure 17D). The results indicated that GRcR/*VEGF-siRNA* could inhibit angiogenesis effectively by suppressing the expression and secretion of VEGF in HUVECs.

### 2.14. Histological and Immunohistochemical Study of Tumor Tissue

**Figure 18:**
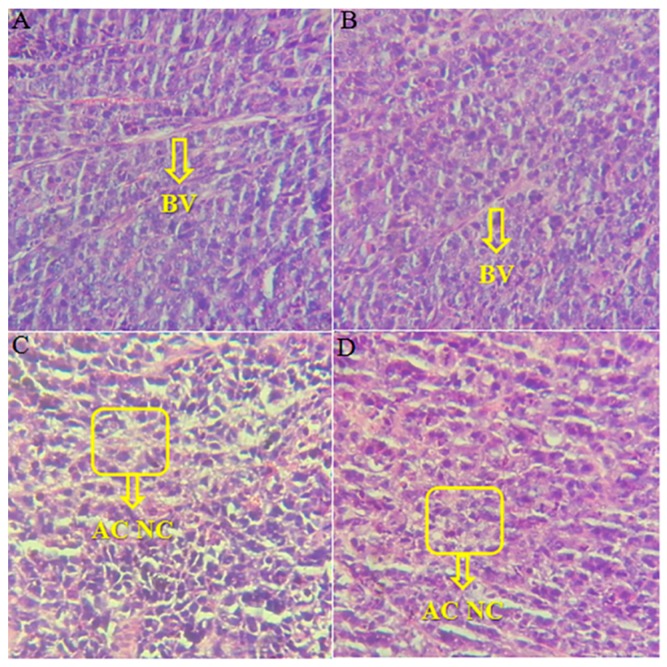
Hematoxylin and eosin (HE) staining of tumor tissue. (**A**) NS group, (**B**) naked *VEGF-siRNA* group, (**C**) GRcR/*VEGF-siRNA* group, and (**D**) DOX group. BV: blood vessels; AC: apoptotic cells; NC: necrotic cells. (Original magnification: 40×)

Hematoxylin and eosin (HE) staining revealed the histological change of tumor tissue after treatment with GRcR/*VEGF-siRNA*. Slices of the NS and naked *VEGF-siRNA* groups are shown in Figure 18A,B. Cytoplasm of normal tumor cells was abundant, and blood vessels could be observed clearly. While in Figure 18C,D, after treatment with GRcR/*VEGF-siRNA* and DOX, hemorrhage and necrosis were observed and the blood vessels were difficult to recognize. The results revealed that GRcR/*VEGF-siRNA* could damage tumor tissue, inhibit the formation of blood vessels, and cause hemorrhage at the tumor area.

**Figure 19:**
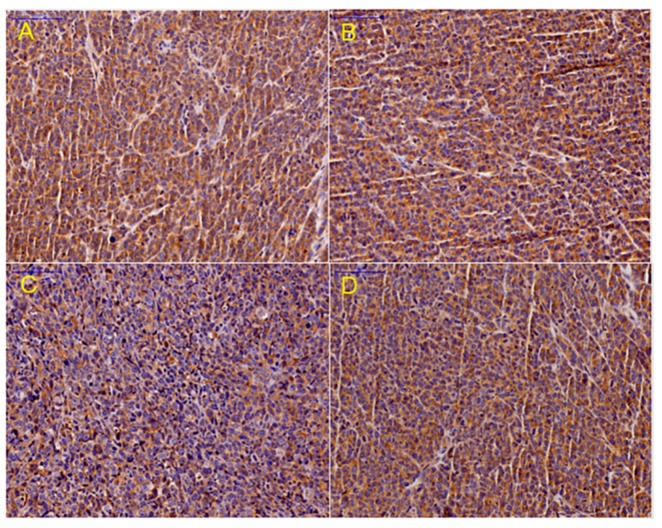
Immunohistochemical analysis of VEGF. (**A**) NS group, (**B**) naked *VEGF-siRNA* group, (**C**) GRcR/*VEGF-siRNA* group, and (**D**) DOX group. (Original magnification: 40×).

An immunohistochemical study was carried out; VEGF protein from the tumor tissue was stained and is shown in yellow. As shown in Figure 19, the expression of VEGF protein in the GRcR/*VEGF-siRNA* group was significantly suppressed; this indicates that the inhibition of the angiogenesis of tumor tissue is related to the downregulation of VEGF protein expression.

**Figure 20:**
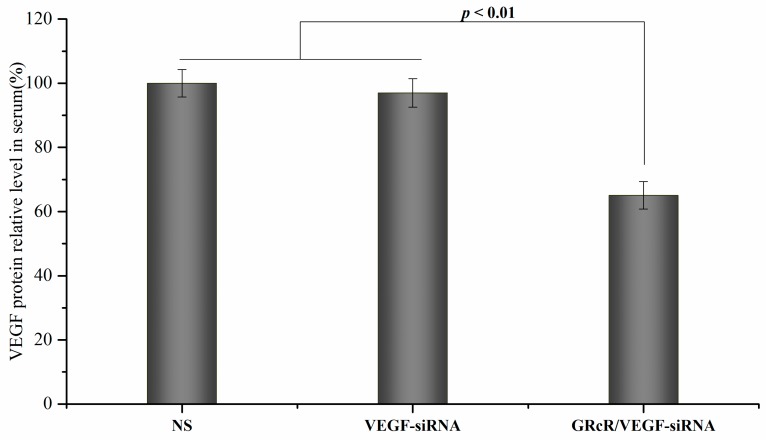
Serum VEGF level ELISA. The data was presented as the mean ± SD, *n* = 5.

Serum VEGF protein level was also evaluated, blood samples from NS, naked *VEGF-siRNA*, and GRcR/*VEGF-siRNA* groups were analyzed using a mouse VEGF ELISA kit (Figure 20). The result showed that the serum VEGF protein level of the GRcR/*VEGF-siRNA* treated group was lower than that of the NS and naked *VEGF-siRNA* groups (43% reduction); it also illustrated that the tumor growth inhibition mechanism was suppressing the expression of VEGF protein.

## 3. Discussion

Graphene oxide (GO) has proven to be a promising nanocarrier in recent years, and has gained attention in many different fields [28]. Modified GO has been used in many pharmaceutical researches as a drug and gene delivery carrier, biosensor, and bioimaging agent [29,30,31].

In this research, we comodified GO with R8 and cRGDfV and successfully prepared a gene carrier, GRcR. The structure, composition, and morphology of the carrier were then studied by FTIR, UV, TEM, and TGA. The composition of the complex was calculated [32,33]: 85.04 wt % GO, 13.05 wt % R8, and 1.91 wt % cRGDfV. More parameters of this carrier were characterized, including the stability, electropositivity, and loading capability. The zeta potential study showed that, after being modified with R8 and cRGDfV, the zeta potential decreased slightly to 29 ± 5.32 mV; the electropositivity could also provide evidence of stability and siRNA loading capability improvement [34]. The stability studies showed that GRcR has good stability in water after seven days at room temperature; the loading capability of siRNA (*w/w* 40:1) was determined by gel retardation experiments. Concurrently, the release profile of *VEGF-siRNA* from GRcR was investigated, and the result showed that the GRcR could be used as a late release carrier in gene delivery systems. 

In vitro studies were carried out, including a cytotoxicity assay, transfection ability assay, and tumor cell inhibitory assay. The results showed that GRcR/*VEGF-siRNA* could be taken-up by tumor cells and cause cell growth inhibition at 30 nM *VEGF-siRNA*. Additionally, the carrier showed no significant cytotoxicity under 150 µg/mL. Further studies have been designed to investigate the tumor cell inhibitory activity of GRcR-*VEGF-siRNA*. RT-PCR and ELISA assays were carried out to investigate the gene silencing effect of the delivery system on mRNA and protein levels. Results showed that the expression of VEGF mRNA and protein were downregulated by 48.22 and 38%, respectively. 

In vivo experiments showed that tumor growth could be inhibited by administrating GRcR/*VEGF-siRNA* intravenously every other day; the tumor inhibitory rate was observed at 53.15%. A fluorescent imaging assay was used to investigate the distribution of GRcR/*VEGF-siRNA*, the results showed that GRcR/*VEGF-siRNA* could concentrate *VEGF-siRNA* at the tumor area after 30-min administration. Further, the histological and immunohistochemical study of tumor tissue revealed that GRcR/*VEGF-siRNA* could inhibit the formation of blood vessels and downregulation of VEGF protein expression by 43%. This result may be due to the fact that cRGDfV had synergistic function with *VEGF-siRNA* to inhibit angiogenesis [35,36].

In summary, GRcR is a comodified carrier designed for gene delivery, it showed both high transfection ability as well as tumor-targeting ability. Further studies are warranted to investigate the pharmacokinetics of the complex and its absorption, metabolization, and excretion pathways.

## 4. Materials and Methods

### 4.1. Chemicals and Reagents

Graphene oxide was purchased from Sigma-Aldrich (St. Louis, MO, USA), Octaarginine (R8) and cRGDfV (Arg-Gly-Asp-Phe-Val) were purchased from GL Biochem Ltd. (Shanghai, China) and Popchem Co., Ltd. (Hefei, China), respectively. The HeLa cell line was obtained from the Chinese Academy of Medical Science tumor cell bank (Beijing, China). Fetal bovine serum (FBS), DMEM medium, HyQ trypsin, and PBS were purchased from HyClone (Logan, UT, USA). *VEGF-siRNA*, FAM-*VEGF-siRNA*, Cy5-*VEGF-siRNA*, and the negative control *VEGF-siRNA* (NC) were purchased from GL Biochem Ltd. (Shanghai, China). Lipo™2000 (Lipo) was purchased from Invitrogen (Carlsbad, CA, USA). Penicillin and streptomycin were purchased from Sigma Chemical (St. Louis, MO, USA). BCA protein kit was purchased from Pierce (Rockford, IL, USA). Human VEGF ELISA Kit and Mouse VEGF ELISA Kit, high Capacity cDNA Reverse Transcription Kit, High Capacity RNA-to-cDNA Kit, TaqMan Gene Expression Master Mix, and TaqMan Gene Expression Assays (VEGF assay, GAPDH assay) were purchased from Thermo Fisher Scientific (Waltham, MA, USA). TRIzol was purchased from Invitrogen. Matrigel was purchased from BD Bio-sciences (New Jersey, USA). All reagents were analytical grade and were used without any further purification. Deionized (DI) water was used in all experiments.

The sequences of mus-*VEGF-siRNA*: 5′-CGAGGCAGCUUGAGUUAAATT-3′ (sense); 5′-UUUAACUCAAGCUGCCUCGTT-3′(antisense).

The sequences of homo-*VEGF-siRNA*:5′-GCGCAAGAAAUCCCGGUAUTT-3′ (sense); 5′-AUACCGGGAUUUCUUGCGCTT-3′ (antisense).

The sequences of NC (negative control): 5′-UUCUCCGAACGUGUCACGUTT-3′ (sense); 5′-ACGUGACACGUUCGGAGAATT-3′ (antisense).

### 4.2. Carboxylation of Nano-Graphene Oxide Sheets (NGOS–COOH)

GO (50 mg) was dispersed in 30 mL of deionized water, the suspension was then sonicated for 30 min, and followed by adding 5 g NaOH and 5 g sodium chloroacetate in ice bath [37,38,39]. Briefly, the suspension was sonicated for 4 h and then stirred at the room temperature for an additional hour to transform –OH groups to –COOH by reacting with sodium chloroacetate. After the preparation formed, the NGOS-COOH solution was centrifuged at 125× *g* for 10 min, the precipitation was separated and rinsed with distilled water for three times. After freeze-drying, NGOS-COOH was stored for further use.

### 4.3. Preparation of GRcR Complex

To increase the electropositivity of the carrier, R8 was immobilized onto the surface of NGOS-COOH [31,40,41]. Briefly, freeze-dried NGOS-COOH (20 mg) was suspended in 20 mL of tridistilled water by sonication for 1 h. After stirring for 30 min, the R8 (60 mg) solution was added into the mixture. The mixture was then stirred at room temperature for another 48 h, followed by centrifuging for three times; the precipitation was freeze-dried and stored for further use. The powder (10 mg) was dispersed into 10 mL of water by sonication for 1 h. Then 0.2 mg of cRGDfV powder was added into the suspension, sonicated, and vortex for 2 h. The mixture was then left at 4 °C overnight, washed with DI water, and centrifuged at 125× *g* for 15 min to remove unbound cRGDfV [42,43]. 

### 4.4. Gel Retardation Assay and Preparation of GRcR/VEGF-siRNA

In order to investigate the loading capacity of the prepared carrier, a gel retardation assay was carried out using *VEGF-siRNA* as the substrate. *VEGF-siRNA* (1 µg) was mixed with GRcR solution at different ratios (*w/w*: 0–40) and incubated at room temperature for 30 min. The complexes were then electrophoresed under 120 V for 30 min (1% agarose gel with 10 mg/mL EtBr) in Tris/Borate/EDTA (TBE) buffer. The results were recorded by UV transilluminator.

### 4.5. Release Profile of GRcR/VEGF-siRNA

The release profile of GRcR/*VEGF-siRNA* was carried out by dialysis experiments using RNase-free centrifugal tubes. FAM labeled *VEGF-siRNA* were used, while *VEGF-siRNA* (500 μL, 100 nM), GO/*VEGF-siRNA*, and GRcR/*VEGF-siRNA* (contain 100 nM *VEGF-siRNA*) were dispersed into Diethyl pyrocarbonate (DEPC) water and placed in dialysis membrane (MWCO: 100 kD), respectively. DEPC water was used as the internal phase, and 5 mL of TE buffer (Tris-HCl (10 mM) and EDTA (1 mM, pH 8.0) was used as the external phase. The dialysis was carried out at 37 °C and the external phase was then collected and replaced with fresh TE buffer at the selected time points. The amount of released of *VEGF-siRNA* was measured by fluorescence spectrophotometer (excitation 492 nm, emission 518 nm); the percentage of cumulatively released *VEGF-siRNA* was calculated according to the standard curve of *VEGF-siRNA*. All experiments were repeated in triplicate.

### 4.6. Cell Culture

HeLa cells were cultured in complete DMEM (10% fetal bovine serum (FBS), 1% penicillin, and streptomycin) at 37 °C in 5% CO_2_ atmosphere. Cells were harvested and seeded into 96-well plates till the confluence reached 70–80%.

### 4.7. Cytotoxicity Assay

HeLa cells were seeded in a 96-well plate at the density of 4 × 10^3^ cells per well. After 24-h incubation, the complete DMEM was replaced with 100 µL of GRcR solution at different concentrations (5–150 µg/mL). DMEM without GRcR was used as the sterile control. All test plates were incubated for 48 h, each well was washed with PBS and 50 µL of MTT (6 mg/mL) were added, followed by incubating for an additional 4 h. After the incubation, the MTT solution was removed and the formazan crystals were dissolved in DMSO (50 µL per well). The absorbance was measured at 570 nm using a multimode plate reader. All experiments were repeated for three times.

### 4.8. Cellular Uptake of GRcR/VEGF-siRNA

To investigate the cellular uptake of GRcR/VEFG-siRNA, FAM labeled *VEGF-siRNA* was used in this experiment. *VEGF-siRNA* was transfected against HeLa cells by GRcR, and HeLa cells were cultured and seeded in confocal dish (diameter 20 mm) at a density of 1 × 10^5^ cells per dish. All test dishes were incubated for 12 h before the experiment. FAM-*VEGF-siRNA* (10 nM) was mixed with test carriers for 30 min at room temperature, then the complexes were added to the cells in the serum free medium. After an additional 6 h incubation, culture medium were replaced with working solution containing 1 µg/mL Hoechst 33342 for 20 min followed by washing with PBS thrice. The results were observed using laser confocal scanning microscopy.

### 4.9. Antiproliferation Assay

HeLa cells was seeded into a 96-well plate at the density of 4 × 10^3^ cells/well and incubated overnight at 37 °C. The medium was then replaced with naked *VEGF-siRNA*, naked NC, Lipo™2000/*VEGF-siRNA*, Lipo™2000/NC, GRcR/*VEGF-siRNA*, and GRcR/NC in serum-free medium. After 6-h incubation, the transfection medium was removed, and 100 µL fresh DMEM medium was added for another 48 h of incubation. The results were evaluated using MTT assay.

### 4.10. Real Time PCR

The VEGF mRNA level of transfected HeLa cells were studied, and amplified by RT-PCR. HeLa cells were placed on 6-wells plates at density of 2 × 10^5^ per well at 37 °C for 24 h, and transfected with naked *VEGF-siRNA* (100 nM), Lipo™2000/*VEGF-siRNA* (100 nM), GRcR/NC (100 nM), and GRcR/*VEGF-siRNA* (100 nM) for 8 h, respectively. Total RNA was extracted by adding 2 mL TRIzol reagent. The RNA concentration was measured by nanodrop spectrophotometer. cDNA was prepared using a High Capacity RNA-to-cDNA Kit, and 2 mL of cDNA was amplified by reverse transcription-polymerase chain reaction (RT-PCR); housekeeping gene GAPDH was used as inner reference. There were three duplicates for each sample, and all results were reported as relative quantity of expression using the ΔΔ*C*_t_ method.

### 4.11. ELISA

HeLa cells were seeded on a 6-well plate at density of 2 × 10^5^ cells per well, incubated for 24 h. After 8-h transfection with 100 nM naked *VEGF-siRNA*, Lipo™2000/*VEGF-siRNA*, Lipo™2000/NC, GRcR/*VEGF-siRNA*, and GRcR/NC the plates were incubated for another 48 h and the supernatants were collected and centrifuged (125× *g*, 4 °C) for 10 min. Total protein concentration in the supernatant were measured using a Bicinchoninic acid (BCA) Protein Assay kit, according to the manufacturer’s instruction, while the amount of VEGF protein in the supernatant was measured using Human VEGF ELISA Kit according to the manufacturer’s instruction. All experiments were repeated three times.

### 4.12. Tumor Growth Inhibition of GRcR/VEGF-siRNA In Vivo

Female BALB/c nude mice (five weeks, 18–20 g) were purchased from Animal Department of Capital Medical University (Beijing Laboratory Animal Center, Beijing, China). The trial was approved by the Institutional Animal Ethics Committee of Capital Medical University (The ethical code: AEEI-2015-064. Date of approval: 25th May 2015). All animal work was performed according to the Health Guidelines of the Capital Medical University. Written informed consent was obtained from all participants.

All mice were housed in sawdust-lined cages at constant temperatures (22–25 °C) and suitable humidity (50 ± 2.0%). Food and water were available ad libitum. Cervical cancer xenografts were made by inoculating 5.0 × 10^6^ HeLa cells subcutaneously at the right armpit. Tumor volume was measured using calipers, and after tumor volumes reached approximately 350 mm^3^, all test candidates were divided into four groups, including normal saline group, naked *VEGF-siRNA* (0.3 mg/kg) group, GRcR/*VEGF-siRNA* (0.3 mg/kg) group, and DOX (2 µmol/kg) group randomly (10 animals each). The test candidates and controls were given to all mice intravenously five times every other day [44,45]. All mice were sacrificed after the last injection, tumors, organs, and blood were harvested and stored for further use.

### 4.13. Tumor Targeting Ability of GRcR/VEGF-siRNA

To investigate the distribution of GRcR/*VEGF-siRNA*, florescent images of each mouse were obtained using the in Vivo Imaging System FX Pro. Cancer-bearing mice were divided into two groups randomly and given Cy5-*VEGF-siRNA* and GRcR/Cy5-*VEGF-siRNA* intravenously, respectively. After mice were sedated using 2% isoflurane, fluorescent sagittal images of each animal were recorded at 0.5, 5, and 8 h [46,47].

### 4.14. Matrigelangiogenesis Assay

In order to investigate the expression of VEGF in GRcR/*VEGF-siRNA* transfected HeLa cells, HUVECs cells (human umbilical vein endothelial cells) were used in the matrigel angiogenesis assay. HeLa cells (3.0 × 10^5^ cells/well) were seeded in 6-well plates and cultured for 12 h, followed by transfecting with GRcR/*VEGF-siRNA* (100 nM) and Lipo^TM^2000/*VEGF-siRNA* (100 nM). Lipo™2000 was used as the positive control, and blank DMEM was used as the negative control. After 8 h of incubation, the culture medium was replaced with fresh DMEM and cultured for another 48 h to collect the supernatants. HUVECs were suspended in the collected supernatants and seeded onto the matrigel in 96-well plates at the density of 6.0 × 10^3^ cells/well, while HUVECs in the fresh DMEM medium served as the blank control (no VEGF). After 6 h of incubation, the cells were observed under inverted microscope.

### 4.15. Histological and Immunohistochemical Observation

To observe histological appearance of tumor tissue under the influence of GRcR/*VEGF-siRNA*, the tumor tissues were collected and fixed in polyoxymethylene. Tumor sections (5 µm) were prepared by slicing embedded tumor tissue in paraffin. All sections were HE-stained and the immunohistochemical test of VEGF performed [48,49].

### 4.16. Statistical Analysis

Data was represented as the mean ± standard deviation (Mean ± SD). Statistical analysis between test candidates groups and control groups were performed using paired-sample *t*-test at the 0.05 and 0.01 levels of probability to determine significance.

## 5. Conclusions

A tumor-targeting *VEGF-siRNA* carrier (GRcR) was designed and prepared successfully in this research to load and deliver *VEGF-siRNA* into cancer cells. GRcR exhibited excellent stability and transfection ability with low cytotoxicity. The covalently linkage of R8 provided a positive surface charge and warranted the *VEGF-siRNA* loading capacity, while cRGDfV modification improved the tumor-targeting ability. The gene slicing studies showed that GRcR could effectively load and deliver *VEGF-siRNA* into cancer cells and interfered with mRNA, suppressing VEGF expression. The in vivo studies also revealed significant tumor growth inhibitory activity and excellent tumor-targeting efficacy. In summary, GRcR was a very promising tumor-targeting gene delivery vector which could be used in gene interfering therapies in cancer treatment.
